# Human NK cell development requires CD56-mediated motility and formation of the developmental synapse

**DOI:** 10.1038/ncomms12171

**Published:** 2016-07-20

**Authors:** Emily M. Mace, Justin T. Gunesch, Amera Dixon, Jordan S. Orange

**Affiliations:** 1 Center for Human Immunobiology, Baylor College of Medicine and Texas Children's Hospital, Houston, Texas 77030, USA; 2 Immunology Graduate Program, Baylor College of Medicine, Houston, Texas 77030, USA; 3 Department of Biochemistry and Cell Biology, Rice University, Houston, Texas 77005, USA

## Abstract

While distinct stages of natural killer (NK) cell development have been defined, the molecular interactions that shape human NK cell maturation are poorly understood. Here we define intercellular interactions between developing NK cells and stromal cells which, through contact-dependent mechanisms, promote the generation of mature, functional human NK cells from CD34^+^ precursors. We show that developing NK cells undergo unique, developmental stage-specific sustained and transient interactions with developmentally supportive stromal cells, and that the relative motility of NK cells increases as they move through development *in vitro* and *ex vivo*. These interactions include the formation of a synapse between developing NK cells and stromal cells, which we term the developmental synapse. Finally, we identify a role for CD56 in developmental synapse structure, NK cell motility and NK cell development. Thus, we define the developmental synapse leading to human NK cell functional maturation.

## Abstract (web summary)

CD56, a splicing variant of NCAM, marks human NK cell differentiation stages. Here the authors show that developing human NK cells form CD56-enriched synapses with stromal cells, and CD56 is critical to promote motility of NK cells that increases with their maturation.

## Graphical abstract

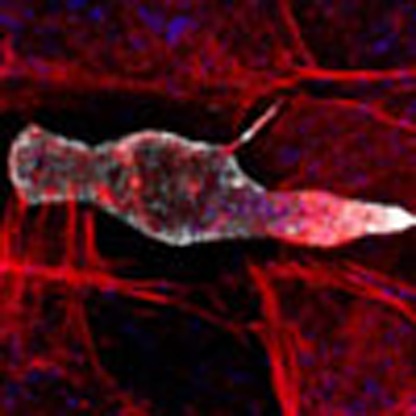

Natural killer (NK) cell development remains a poorly understood process. The site(s) in which NK cells may develop and the environment within these sites that shape functional and phenotypic maturation remain a mystery. Although bone marrow generates CD34^+^ precursors that give rise to NK cells, there are potentially multiple sites for extramedullary NK cell development, including liver, gravid uterus and secondary lymphoid tissue^1^. Pioneering work on NK cell intermediates in human lymph node described five distinct phenotypes corresponding to linear stages of NK cell development^2^,^3^. While this has been refined in light of the discovery and characterization of innate lymphoid cells, the molecular basis for the supportive nature of the lymph node microenvironment (or similarly nurturing environment) is not known.

Human NK cells can be generated from CD34^+^ precursors in multiple ways, including feeder cell-free and -dependent systems^4^. The most efficient system, that results in the significant expression of killer cell immunoglobulin-like receptors (KIR) on mature NK cells, utilizes cytokines and the EL08.1D2 stromal cell line. While other stromal cell lines aid in expanding populations of NK cells, EL08.1D2 are most efficient in their ability to significantly induce terminal maturation^5^. Further, their effectiveness requires direct contact with EL08.1D2 cells^5^. The contact-dependent factors that make these cells uniquely able to support this terminal stage of NK cell development are unknown, however suggested factors include stromal ligands such as Notch, soluble factors such as Wnt or cytokines, or extracellular components such as heparin^4^,^6^,^7^,^8^.

Human peripheral blood contains two primary NK cell subsets (aside from uncommitted CD34^+^ precursors found at low frequency): CD56^bright^ and CD56^dim^. Each represents a distinct phenotypic and functional subset, with a well-described pattern of expression of cell surface and intracellular molecules as well as functional capabilities. It is thought that these stages represent linear stages of terminal maturation, with CD56^bright^ NK cells becoming CD56^dim^ NK cells, most likely in secondary lymphoid tissue^2^. The most convincing evidence for this is the appearance of CD56^bright^ NK cells before CD56^dim^ NK cells both following hematopoietic stem cell transplant and in *in vitro* culture systems^9^,^10^,^11^. Freshly isolated CD56^bright^ NK cells can become CD56^dim^ NK cells in culture^9^, namely in response to FGFR1-mediated signalling, however this is poorly understood in the context of a signal that shapes this response in lymphoid tissue.

Despite its importance as a phenotypic marker of human NK cells, the role of CD56 in NK cell biology remains mysterious. A member of the Ig superfamily, NCAM can be expressed in several isoforms, with CD56 the 140 kDa isoform^12^. While signalling through NCAM molecules results in neurite outgrowth and cell motility on neural cells^13^,^14^,^15^, signalling through CD56 on human NK cells has not been described. The absence of orthologous NCAMs on murine NK cells has made it difficult to determine a requirement for CD56 in function or development. The identification of CD56 as NCAM-140 led to the hypothesis that it played a role in lymphocyte adhesion^16^, however subsequent studies showed that it was not required for cytotoxic function or homophilic interactions with target cells^12^. The role of FGFR1 in CD56^bright^ to CD56^dim^ transition implicates CD56 in this process, as NCAM–FGFR1 interactions in neural cells are well described, however this was not directly tested^9^.

Two-photon imaging of NK cells labelled in murine lymph node reveals a highly motile phenotype, with interactions between NK cells and dendritic cells (DCs) as well as stroma and collagen fibres^17^,^18^. In addition, fixed-cell sections of human LN show CD56^bright^ NK cell colocalization with DCs in the T-cell region, an interaction that likely results in the stimulation and subsequent proliferation of NK cells by DCs, namely through IL-12 and IL-15 (ref. 19). The immunological synapse was first described formally with regards to the T cell–APC synapse^20^,^21^ and the term was coined based on the ‘specialized junction, cell polarization and positional stability' of the T cell–APC interface, which resembled those found in neural cell synapses^20^. The definition of an immunological synapse has since been modified to include NK cell activating and inhibitory synapses^22^,^23^ and NK–DC synapses^24^. The expansion of the term has allowed for inclusion of non-secretory synapses, yet all still follow Dustin's original criteria which can be formally defined as (1) adhesion, (2) polarity and (3) signalling (originally defined as Ca^2+^) and resulting function^25^,^26^. While immune cell development, specifically NK cell development, is a contact-dependent process, there has yet to be the identification of an immunological synapse in this context.

Given the poorly understood molecular requirements for NK cell development, we sought to define the contact-dependent processes that occurred in a system that specifically promoted the terminal maturation of human NK cells with the particular consideration of there being a specialized immunological synapse to promote development. We designed a model in which we subject freshly isolated human NK cells undergoing direct interactions with developmentally supportive EL08.1D2 stromal cells to high-resolution live-cell confocal imaging and rigorous quantitative analysis. We found that human NK cells exhibit unique, stage-specific patterns of motility on stromal cells. This includes migration punctuated by arrest and conjugation through a CD56 and CD62L-enriched platform that leads to F-actin accumulation, tyrosine phosphorylation and calcium flux. We propose that the contact-dependent processes required for NK cell maturation occur through this structure, which we have named the developmental synapse. We show that NK cell motility increases through development and correlates with expression of CD56, which supports migration on developmentally supportive stroma and downstream maturation. Therefore, we identify the contacts formed between NK cells and developmentally supportive stromal cells through development. These contacts include distinct, CD56-driven migratory behaviours but importantly include the developmental synapse, a bona fide immunological synapse that shapes human NK cell functional maturation.

## Results

### NK cell subsets show differential motility on stromal cells

**Figure 1:**
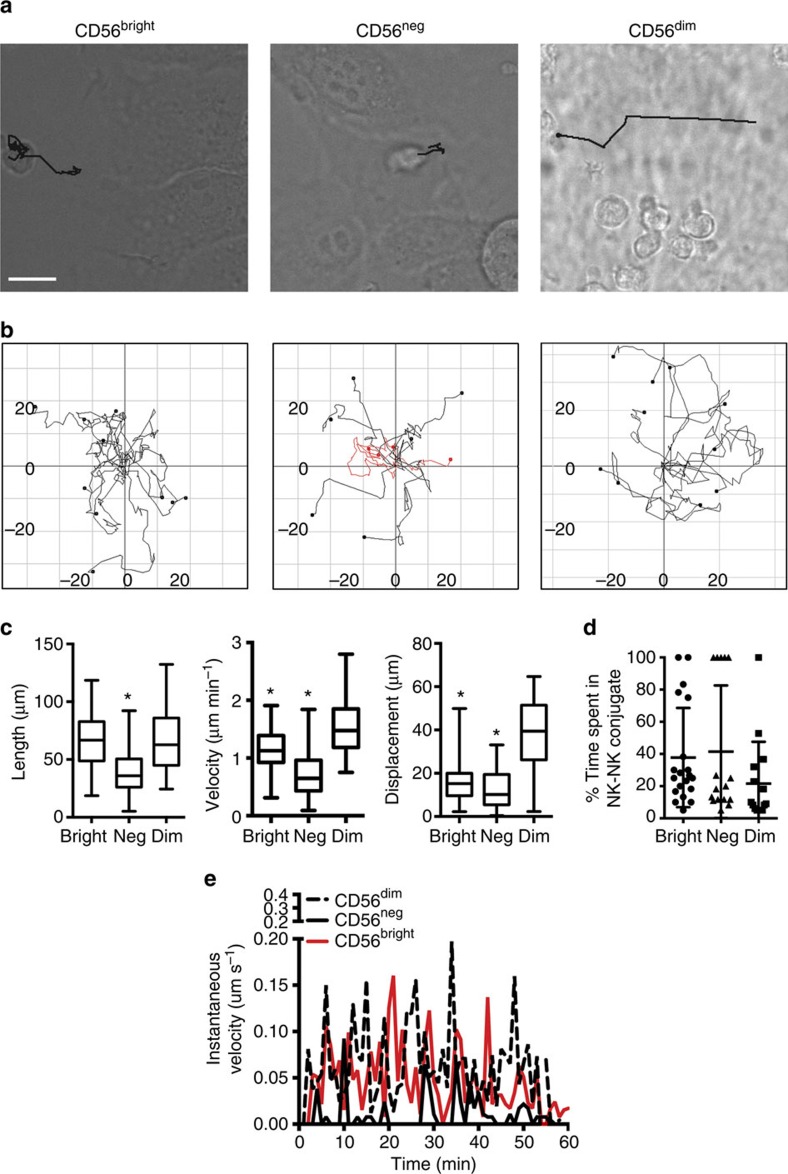
Human NK cell subsets show differential motility on developmentally supportive stroma. NK cell subsets were purified from peripheral blood by FACS sorting then incubated on EL08.1D2 stromal cells for 4 h before initiation of imaging. Cells were imaged by confocal microscopy for 30 min at two frames per minute and tracked using the manual tracking plug-in (FIJI) from start to end of imaging. (**a**) Representative cell from each condition is shown with its track from 0 to 30 min. Scale bar, 10 μm. See also Supplementary Movies 1–3. (**b**) Tracks from ten cells per condition were overlaid using the Chemotaxis tool (FIJI). Tracks with mean velocity slower than 1 μm per minute are shown in red. Units=μm. (**c**) Track length, displacement and velocity were measured from live-cell confocal images (*n*=96 [CD56^bright^], 82 [CD56^neg^]; 36 [CD56^dim^], three independent experiments). Data are represented as mean±s.d. *P<0.05 by Student's unpaired *t*-test. *P*<0.0001 by ordinary one-way ANOVA. *P<0.05 by *post hoc* Tukey's comparison with CD56^dim^ condition. (**d**) NK cells were incubated on EL08.1D2 stroma and imaged 2 frames per min. Duration of contact between individual NK cells and stroma or other NK cells was measured and the percentage of time spent in NK–NK conjugates was calculated (*n*=15–20 per condition from three independent experiments). (**e**) Instantaneous velocity of one cell from the CD56^bright^ (red) CD56^neg^ (black) and CD56^dim^ (black, dashed) populations.

To determine the nature of the interactions between human NK cells and developmentally supportive stroma, we purified NK cell subsets and defined their behaviour using confocal microscopy over 30 min of imaging. We initially chose the CD56^bright^ and CD56^dim^ NK cell subsets as these were accessible from peripheral blood. We also included in our analysis CD56^neg^ NK cells, defined as being CD56^low^CD3^−^CD16^+^CD57^+^ KIR^+^ (ref. 27). In addition to having unique functional properties, each of these subsets represents a distinct developmental population with a well-defined phenotype. Each had a distinct pattern of motility on stroma, with CD56^dim^ NK cells moving in rapid, linear tracks, CD56^bright^ NK cells moving in shorter, multi-directional tracks and CD56^neg^ NK cells demonstrating minimal migration (Fig. 1a; Supplementary Movies 1–3). To ensure that antibody used to isolate subsets was not affecting motility, CD56^bright^ cells were rested for up to 72 h post sorting before initiation of imaging, which showed no significant effect of the time of rest on track length, displacement or velocity (Supplementary Fig. 1). For subsequent experiments, sorted cells were rested 4–8 h before incubation on stromal cells unless otherwise stated. To quantify motility, we tracked individual cells throughout the time of imaging. Tracks were plotted (Fig. 1b) and directionality was calculated; both CD56^dim^ and CD56^bright^ NK cells had non-directed movement, however CD56^dim^ cells appeared to have greater persistence of direction relative to that of CD56^bright^ cells. Track length, velocity and displacement were calculated and in aggregate substantiated the individually observed cell motilities (Fig. 1c). Specifically, both CD56^bright^ and CD56^dim^ NK cells had significantly longer track lengths (66±22.1 μm; 67.8±27.4 μm) than CD56^neg^ NK cells (39.5±19.6 μm), however the greater displacement of CD56^dim^ NK cells reflected their longer, more persistent tracks, accompanied by a significantly greater velocity (1.6±0.5 μm min^−1^) than the other two subsets (1.1±0.4 μm min^−1^; 0.7±0.4 μm min^−1^, respectively for CD56^bright^, CD56^neg^). Although NK–NK interactions were observed, these were relatively transient and the amount of time spent in homotypic interactions was not significantly different between subsets, suggesting that differences in motility were not due to differences in inherent adhesion properties (Fig. 1d). Finally, the instantaneous velocity of CD56^bright^, CD56^dim^ and CD56^neg^ NK cells were calculated. This showed that the increased velocity observed in CD56^bright^ NK cells was a function of both less time spent in arrest and greater velocity at time points in motion than CD56^neg^ NK cells, whereas the CD56^dim^ NK cell had minimal times of arrest and higher peak velocities than the other subsets (Fig. 1e). Taken together, the distinct phenotypic and functional NK cell subsets have distinctive patterns of motility on the stromal cells that promote their development.

### NK cells conjugate via CD56- and CD62L-enriched structures

**Figure 2:**
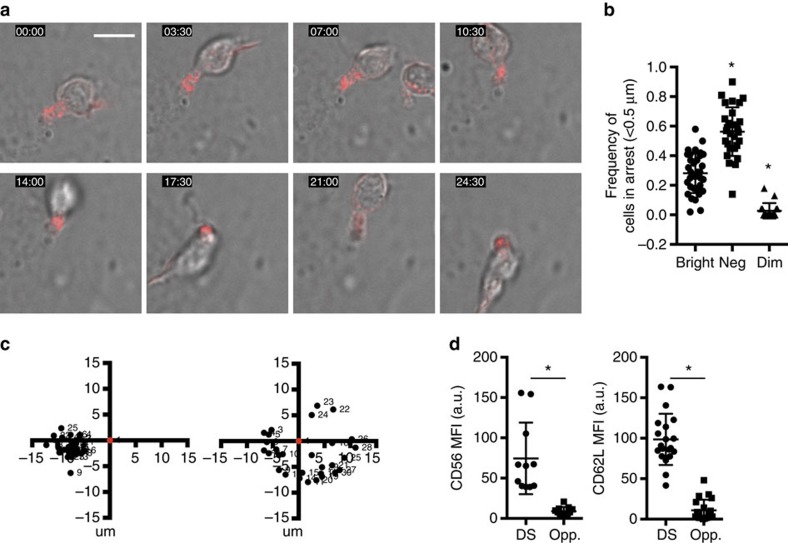
Human NK cells conjugate to stromal cells through a CD56- and CD62L-enriched structure. CD56^bright^ NK cells were purified and incubated on EL08.1D2 stromal cells for 4 h. Cells were imaged as in Fig. 1 in the presence of anti-CD56 (red) antibody. (**a**) A single CD56^bright^ NK cell highlights polarization of CD56 and adhesion to stromal cells. Scale bar, 10 μm. See also Supplementary Movie 4. (**b**) Cells were tracked and the frequency of cells in arrest for each condition was measured (*n*=30). Data are represented as mean±s.d. *P*<0.0001 by ordinary one-way ANOVA. *P<0.05 by *post hoc* Tukey's comparison with CD56^bright^ condition. (**c**) Degrees of rotation of a CD56^bright^ NK cell incubated on anti-CD56 antibody (left) or EL08.1D2 stromal cells (right) and imaged with confocal microscopy. The anchoring point is shown in red and consecutive live-cell frames are shown in black. (**d**) Accumulation of CD56 (left) or CD62L (right) in CD56^bright^ NK cells at the point of contact (DS) or distal site (opp.) when incubated on EL08.1D2 stromal cells and imaged by live confocal microscopy. Data are represented as mean±s.d. *P<0.05 by Student's unpaired *t*-test.

NK cells migrating on developmentally supportive stroma adopted a broad leading edge and a uropod-like trailing end. Surprisingly, however, when CD56^bright^ NK cells were found in arrest, they were conjugated to stromal cells with firm adhesion and a polarized phenotype (Fig. 2a). The addition of fluorescently labelled antibody to CD56 itself, or CD62L, described the polarized accumulation of both these molecules at the site of adhesion and our live-cell imaging showed consistent polarization of conjugated CD56^bright^ NK cells (Fig. 2a; Supplementary Movie 4). We measured the relative amount of time each subset spent in arrest and found that CD56^neg^ NK cells spent the majority of time in arrest (0.56±0.16) and CD56^dim^ NK cells spent a significant amount of time migrating, and therefore not in arrest (0.03±0.05). CD56^bright^ NK cells however, frequently alternated between the two states of arrest and motility with an aggregate intermediate mean arrest time (0.28±0.13) (Fig. 2b). Measurement of the free end of the cell relative to the tethered point of attachment confirmed almost 360 degrees of rotational freedom in a CD56^bright^ NK cell tethered by its uropod to stromal cells (Fig. 2c). The site of contact of the CD56^bright^ NK cells to the stroma appeared not to be incidental as quantification of the intensity of CD56 and CD62L at the site of tethering demonstrated a significant accumulation of both proteins at the intercellular contact (Fig. 2d). We tentatively termed this intercellular contact created by arrested NK cells a ‘developmental synapse' (DS).

### The developmental synapse is a site of signalling

**Figure 3:**
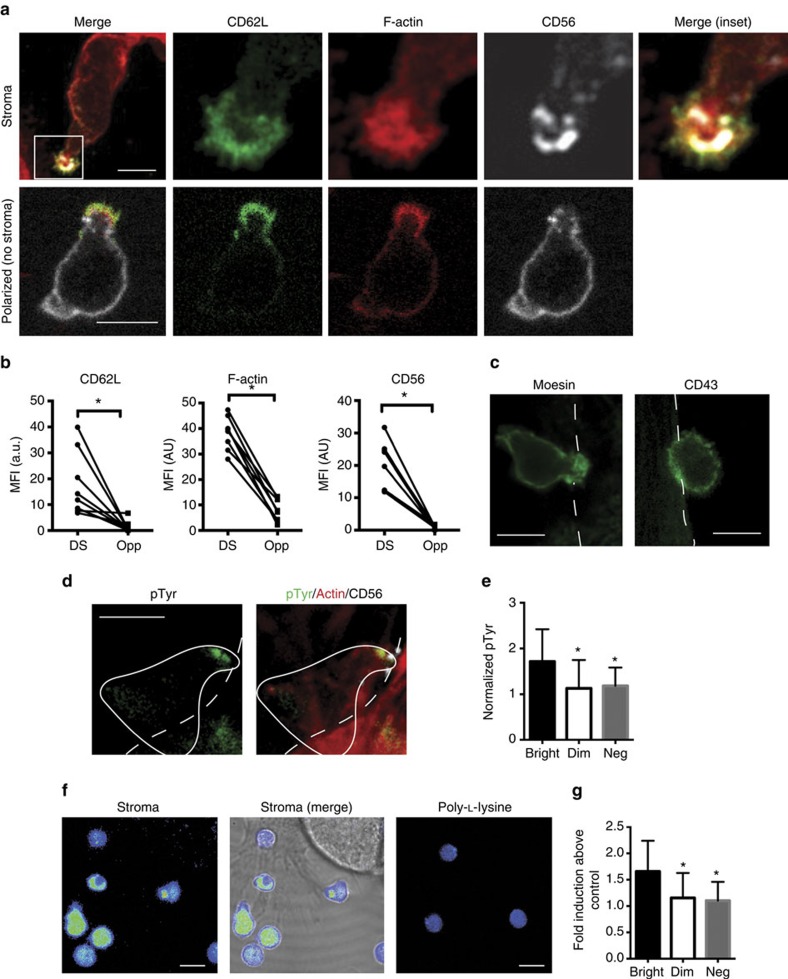
Fixed-cell confocal microscopy defines the DS. (**a**) CD56^bright^ NK cells were incubated on EL08.1D2 stromal cells (top) or gelatin-coated imaging chambers (bottom) for 2 h then fixed, permeabilized and stained for proteins of interest as indicated. Images were acquired by confocal microscopy and analysed as described in Methods. Scale bar, 5 μm. (**b**) Density of CD56, F-actin and CD62L were calculated from confocal microscopy images by measuring fluorescence intensity at the DS or opposite side (opp.). *n*=13 from one experiment representative of three independent repeats. *P<0.05 by Student's unpaired *t*-test. (**c**) CD56^bright^ NK cell tethered to a stromal cell (outlined in dashed line) with CD43 (right) or moesin (left) (green). Scale bar, 5 μm. (**d**) CD56^bright^ NK cell (outlined in solid line) tethered to a stromal cell (outlined in dashed line) with phospho-tyrosine (green), F-actin (red) and CD56 (grey) detected. Scale bar, 3 μm. (**e**) Accumulation of pTyr staining at the DS in purified CD56^bright^, CD56^dim^ or CD56^neg^ NK cells conjugated to stromal cells as in **c**. Values are normalized to the distal portion of the cell body as described in Methods. *n*=40 cells per condition. Data are represented as mean±s.d. *P<0.0001 by Student's unpaired *t*-test compared with CD56^bright^. (**f**) Representative field showing CD56^bright^ NK cells adhered to stromal cells (left and center, with brightfield) or poly-L-lysine (right) with heat map intensity of Ca^2+^ flux as detected by Indo1-AM. Scale bar, 10 μm. (**g**) Ratio of 485/530 nm intensity (normalized to mean ratio of poly-L-lysine control) of purified CD56^bright^, CD56^dim^ or CD56^neg^ NK cells on stroma. Data are represented as mean±s.d. *P<0.0001 by Student's unpaired *t*-test compared with CD56^bright^. *n*=170 cells per condition.

To better define DS components and their localization at high resolution, we performed fixed-cell confocal microscopy of CD56^bright^ NK cells conjugated to EL08.1D2 stromal cells and visualized CD56, CD62L as well as F-actin owing to its central role in establishing immunological synapses (Fig. 3a, top panel). We consistently observed accumulation of CD56 and CD62L and enrichment of F-actin at the DS (Figs 3a,b) in a pattern reminiscent of the uropod of polarized cells in the absence of stroma, which is shown in Fig. 3a (lower panel). Further, we observed accumulation of the uropod markers CD43 and moesin at the DS (Fig. 3c), suggesting the DS may be a uropod-derived structure.

Since a major function of immunological synapses is signal generation, we performed fixed-cell imaging as above with antibody specific for phosphorylated tyrosine which showed distinct punctae of phospho-tyrosine co-localized with F-actin and CD56 at the DS (Fig. 3d). Analysis of CD56^neg^ and CD56^dim^ NK cells conjugated to stroma showed that the accumulation of phospho-tyrosine was found solely significantly in the CD56^bright^ subset (Fig. 3e). Similar patterns of transient arrest, signalling and migration as those observed in CD56^bright^ NK cells have been described in T cells migrating on thymic epithelium, wherein the periods of arrest are associated with calcium flux^28^. To determine if migration and/or tethering of NK cells resulted in calcium flux, we labelled CD56^bright^ NK cells with Indo1-AM and performed live-cell imaging and analysis as above on EL08.1D2 stromal cells or poly-L-lysine (as a negative control) (Fig. 3f). Analysis of >150 cells per condition showed intracellular calcium elevation in those cells conjugated to stroma for 60 min (Fig. 3g). Similar analyses of CD56^dim^ and CD56^neg^ NK cells showed that the significant increase of Ca^2+^ following incubation on stroma was unique to the CD56^bright^ subset (Fig. 3g). While conjugation with stroma led to elevated Ca^2+^, we did not observe a correlation with times of arrest (data not shown). Our data show that the DS may be derived from uropod components, however its firm adhesion, polarization and signalling suggest it is a true immunological synapse.

### CD56^bright^ NK cell motility is dependent on CD56

**Figure 4:**
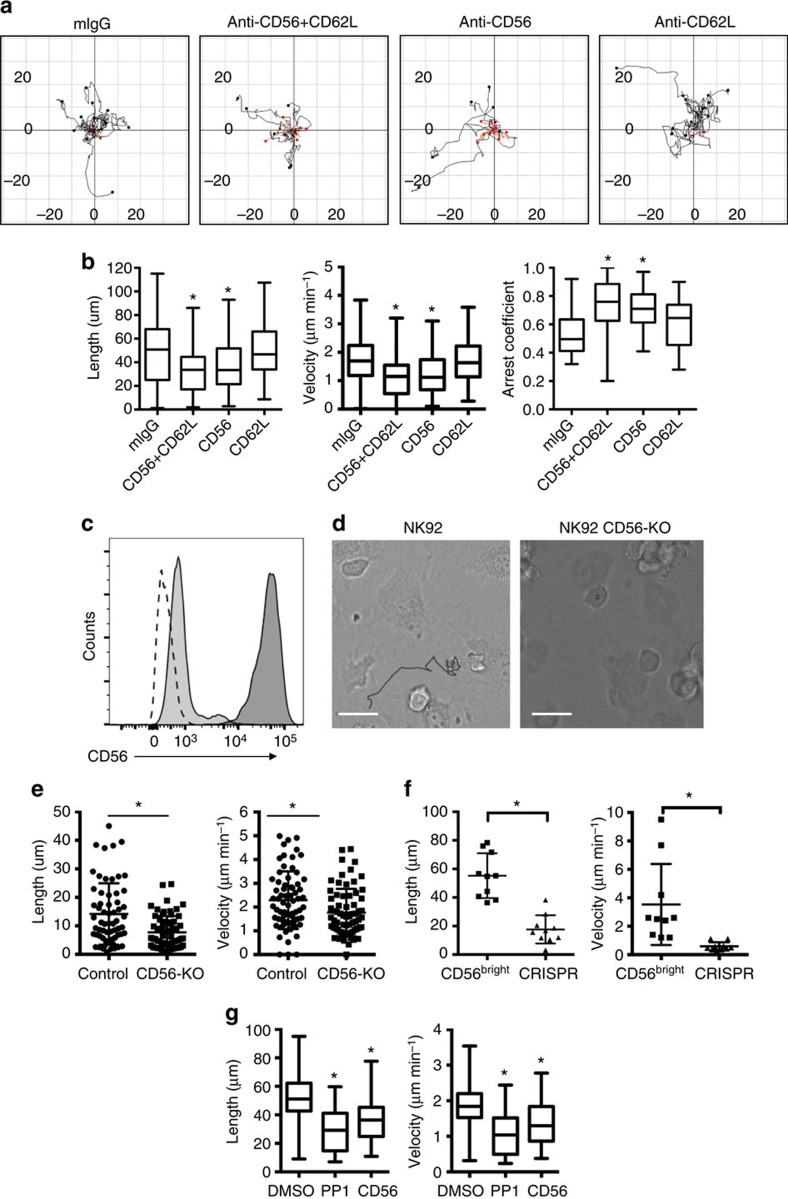
NK cell motility is dependent upon CD56 and Src kinase signalling. CD56^bright^ NK cells were purified and pre-incubated in the presence of anti-CD56 and/or CD62L blocking antibody or mouse IgG as a negative control then incubated on EL08.1D2 stromal cells for 4 h. Cells were then imaged for 30 min (2 frames per minute). (**a**) Cells were tracked with Manual Tracking (FIJI) and tracks with velocity of <1 μm min^−1^ are shown in red. *n*=10 from one representative experiment. (**b**) Length, velocity and arrest coefficient were measured from manually tracked cells. *n*=70 from three independent experiments. Data are represented as mean±s.d. *P*<0.0001 by ordinary one-way ANOVA. *P<0.05 by *post hoc* Tukey's comparison with mIgG condition. (**c**) Expression of CD56 on NK92 CD56-KO cell lines was measured by flow cytometry. Isotype control is shown as a dashed line, wild-type NK92 are represented by the dark histogram and CD56-KO is the light histogram. (**d**) NK92 (wild type, left, or CD56-KO, right) were visualized by live-cell confocal microscopy for 60 min on EL08.1D2 stromal cells and tracked as in Fig. 1. Representative cell from each condition is shown with its track from 0 to 60 min overlaid. See also Supplementary Movies 5 and 6. Scale bar, 10 μm. (**e**) Track displacement and velocity were measured for one representative experiment of three independent repeats described in (**d**) *n*=70. Data are represented as mean±s.d. *P<0.05 by Student's unpaired *t*-test. (**f**) Purified CD56-KO cells derived from CD56^bright^ *ex vivo* NK cells were visualized by live-cell microscopy for 30 min on EL08.1D2 stromal cells and track length and velocity were measured. *n*=10 from one representative experiment of three. Data are represented as mean±s.d. *P<0.05 by Student's unpaired *t*-test. (**g**) CD56^bright^ NK cells were pre-incubated with PP1 Src kinase inhibitor, anti-CD56 blocking antibody or DMSO as a vehicle control then imaged and tracked. *n*=52 from three independent experiments. Data are represented as mean±s.d. *P*<0.0001 by ordinary one-way ANOVA. *P<0.05 by *post hoc* Tukey's comparison with DMSO condition.

NCAM-1 (CD56) has been shown to facilitate migration of other non-hematopoietic cell types^13^. Similarly, CD62L (L-selectin) plays a role in cellular homing to lymph nodes^29^. To determine the role that these proteins played in NK cell motility we pre-incubated NK cells with blocking antibody to CD56, CD62L or both, or mouse IgG as a negative control. Cells were then incubated on stromal cells, tracked, and tracks measured as in Fig. 1 (Fig. 4a). Anti-CD56 blocking, in particular, had a significant effect on the velocity and track length of CD56^bright^ NK cells (Fig. 4b). While blocking CD62L did not significantly decrease motility, the use of both blocking antibodies together had a greater effect on track length and velocity. Blocking one or both receptors also increased the amount of time NK cells spent arrested on stroma (Fig. 4b), suggesting that although they play a role in motility, these receptors are not required for adhesion to stromal cells.

To confirm the role that CD56 functions in NK cell motility via the DS, we performed CRISPR-Cas9-mediated deletion of CD56 on the NK92 cell line (Fig. 4c). Loss of CD56 did not affect other adhesion and activation markers expressed by NK92 cells, including CD18 and CD2 (Supplementary Fig. 2). To test the effect of loss of CD56 on motility we performed live-cell confocal imaging of NK92 (parental and CD56-KO) on EL08.1D2 stromal cells (Fig. 4d). While CD56-KO NK92 retained the ability to adhere to stroma, track length and speed of NK92 CD56-KO tracks were significantly reduced compared with parental NK92 (Fig. 4e; Supplementary Movies 5 and 6). Finally, we performed CRISPR-Cas9-mediated deletion of CD56 on freshly isolated CD56^bright^ NK cells and performed live-cell confocal microscopy and analysis. Consistent with blocking experiments and CD56-KO cell lines, CD56-deleted CD56^bright^ NK cells had significantly reduced track length and velocity from mock-transfected controls (Fig. 4f).

Src family kinases signal from NCAM-1 to promote motility in non-immune cells^30^. As seen in Fig. 4g, the addition of Src kinase inhibitor PP1 reduced CD56^bright^ NK cell track length and velocity significantly when compared with vehicle control. Anti-CD56 blocking, as a positive control, had a similar effect as PP1 treatment. Therefore, CD56, Src family kinases and, to a certain extent CD62L, are required for the motility of CD56^bright^ NK cells on developmentally supportive stromal cells and are engaged through a specific synapse formed between the two cell types.

### Human NK cells show increasing motility with maturation

Given the differences in motility observed between the three developmentally distinct NK cell subsets, we sought to characterize the relationship between NK cells and stromal cells at earlier developmental stages. To do so, we utilized two approaches, namely the isolation of NK cell precursors from human secondary lymphoid tissue and the *in vitro* generation of NK cells from CD34^+^ precursors isolated from peripheral blood.

**Figure 5:**
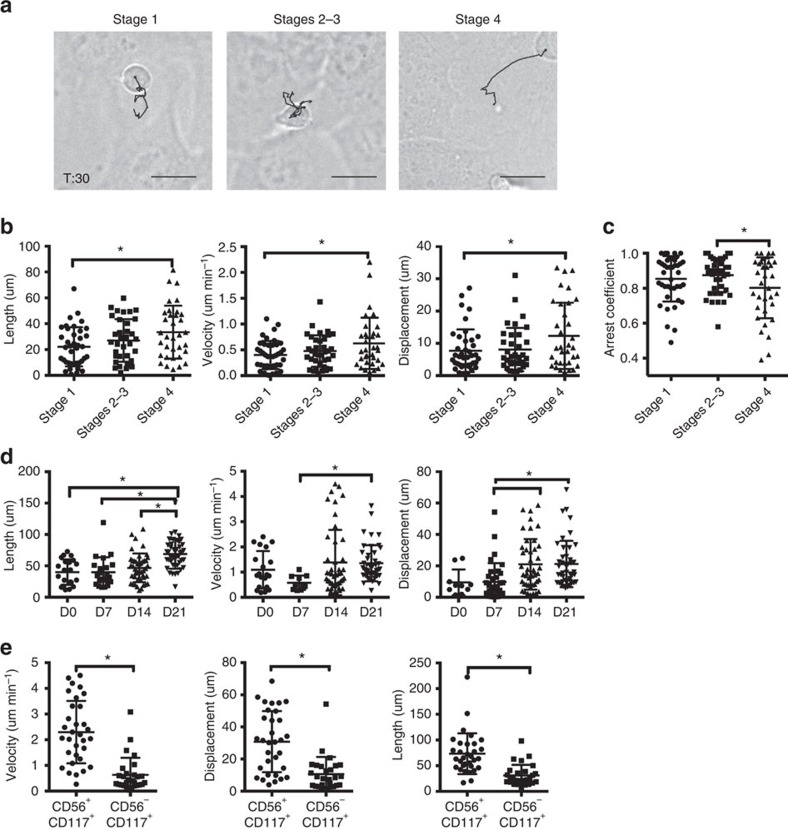
NK cell motility increases with progressive maturation. Developing NK cells were purified from human tonsils as described in Materials and Methods. Following isolation, cells were incubated on EL08.1D2 stromal cells for 4 h then imaged and tracked as in Fig. 1. (**a**) Representative cell from each condition is shown with its track from 0 to 30 min overlaid. Scale bar, 10 μm. (**b**) Length, velocity and displacement were calculated as in Fig. 1 (*n*=34–41 from three independent experiments). *P*<0.0001 by ordinary one-way ANOVA. *P<0.05 by *post hoc* Tukey's comparison. (**c**) Arrest coefficient was calculated as the frequency of cells in arrest (<1 μm track distance) at cumulative time points over the course of imaging. *n*=34–41 from three independent experiments with three separate donors. *P<0.05 by Student's unpaired *t*-test. (**d**) CD34^+^ hematopoietic stem cells were purified from peripheral blood and differentiated to become NK cells as described in Methods. At the time points indicated cells were removed from culture and re-incubated on fresh EL08.1D2 stromal cells for 4 h before imaging. *n*=60 from 3 independent experiments. *P*<0.0001 by ordinary one-way ANOVA. *P<0.05 by *post hoc* Tukey's comparison. (**e**) Tracks from all *in vitro* derived NK cells were pooled and track length, displacement and velocity were calculated for cells expressing CD56 or CD117 as detected by antibody staining at the time of imaging. *n*=45 from three independent experiments. All data are represented as mean±s.d. *P<0.05 by Student's unpaired *t*-test.

We designed a FACs sorting strategy based on reported stages of NK cell development^3^ and isolated Stage 1 NK precursors (lin^−^CD34^+^CD117^−^CD94^−^), a Stage 2/3 combined population (lin^−^CD34^+/−^CD117^+^CD94^−^) and Stage 4 NK cells (lin^−^CD34^−^ CD117^+/−^CD94^+^) from freshly resected tonsils (Supplementary Fig. 3). We then incubated these cells on stromal cells and quantified their motility (Fig. 5a). While Stage 1 NK cells had limited motility (0.4±0.27 μm min^−1^ velocity, 7.7±6.6 μm displacement, 23.8±16.7 track length), Stage 4 NK cells had the motility and displacement more similar to what we had previously observed in CD56^bright^ NK cells (0.6±0.5 μm min^−1^ velocity, 12.3±10.3 μm displacement, 33.5±20.5 track length). The Stage 2/3 population, which has heterogeneous expression of CD56, had an intermediate length, displacement and velocity (0.5±0.3 μm min^−1^ velocity, 8.1±6.6 μm displacement, 28.7±19.1 track length) (Fig. 5b). In addition, the relative frequency of cells in arrest decreased significantly between Stages 2/3 and 4 (Fig. 5c). To further identify differences amongst NK cell developmental intermediates and avoid any potential effects of FACs sorting upon intermediates, we derived NK cells *in vitro* from CD34^+^ precursors. By isolating cells at days 7, 14 and 21 and performing imaging and tracking we determined that, similar to our results with NK cell precursors isolated from tissue, increasing maturation resulted in increasing track length, velocity and displacement (Fig. 5d). During *in vitro* differentiation, expression of CD117 precedes that of CD56 and is detectable at day 7, with day 14 cultures containing both CD56^+^CD117^+^ and CD56^−^CD117^+^ cells, which are largely CD56^+^CD117^+^ by day 21 (Supplementary Fig. 4). At day 21, cells are predominantly CD56^bright^ (Stage 4) (Supplementary Fig. 4), with final, terminal maturation to CD56^dim^ (Stage 5) not occurring until days 28–35 (ref. 5). By comparing motility of CD56^+^ (more mature) versus CD56^−^ (less mature) CD117^+^ cells isolated between days 7 and 21 of culture, we found that expression of CD56 corresponded to a significantly greater track length, velocity and displacement (Fig. 5e). Thus, NK developmental intermediates with the greatest CD56 expression demonstrated the greatest motility on developmentally supportive stroma regardless of their stage of maturation.

### Stromal cells support expression of CD62L on human NK cells

**Figure 6:**
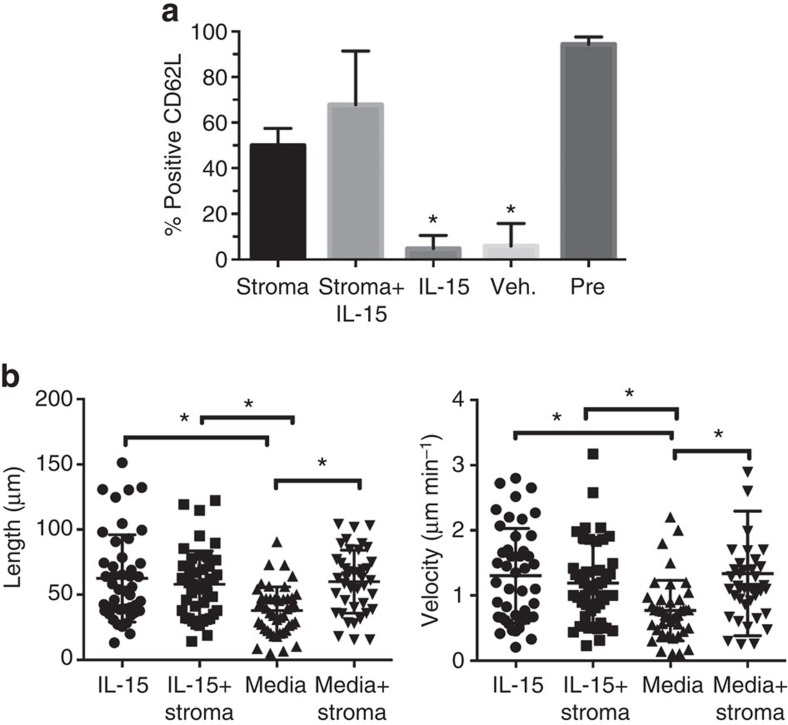
CD62L expression on mature NK cells is reduced in the absence of EL08.1D2 stromal cells. CD56^bright^ NK cells were purified and incubated on EL08.1D2 stromal cells or in uncoated tissue culture dishes for 3 days in the presence of 5 ng ml^−1^ IL-15 or vehicle control. (**a**) Percentage of NK cells expressing CD62L as determined by FACS (*n*=3 wells from three independent experiments for each condition) *P<0.05 when compared to stroma+IL-15 condition by unpaired Student's *t*-test. (**b**) Following incubation cells were harvested and incubated on fresh EL08.1D2 for 4 h before the initiation of imaging. Cells were imaged and tracked as in Fig. 1. *n*=45 from two independent experiments. *P*<0.0001 by ordinary one-way ANOVA. *P<0.05 by *post hoc* Tukey's comparison. All data are represented as mean±s.d.

To identify contact-dependent effects of stromal cell interactions on the expression of NK cell developmental markers we performed flow cytometry phenotyping of CD56^bright^ NK cells following incubation in the presence or absence of EL08.1D2 stromal cells for 72 h. Interestingly, the presence of stromal cells maintained the density of CD62L expression on CD56^bright^ NK cells following 3 days of culture. CD62L on NK cells in direct contact with EL08.1D2 stromal cells was expressed at levels comparable to those observed in freshly isolated cells (Fig. 6a, Supplementary Fig. 5), whereas those maintained in the absence of stroma had significantly decreased CD62L expression. This effect was independent of the presence of IL-15, although IL-15 did increase CD56 expression as previously described^31^. Further, surface density of other markers was not affected (CD117, CD94, CD16 and CD57), suggesting specificity of this effect for CD62L. To correlate the observed effect on CD62L with motility, following culture cells were incubated on fresh stromal cells and then imaged and tracked. The induced loss of CD62L expression resulted in a decrease in track length and velocity comparable to that observed in the presence of CD62L blocking antibody (Fig. 6b). Furthermore, the presence of IL-15 resulted in an increase in motility, likely due to the increased surface density of CD56. Previous studies have identified FGFR1 as a ligand for human CD56, and NCAM molecules are known to engage in homotypic interactions^9^. Flow cytometry analysis of stromal cell lines showed that they expressed detectable levels of mouse NCAM (bound by mouse-specific anti-NCAM antibody) but were not bound by anti-human CD56 antibody used for cell sorting (Supplementary Fig. 6A). This was further confirmed by confocal microscopy showing localization of NCAM to sites of contact between stroma and human NK cells (Supplementary Fig. 6B). Western blot analysis of FGFR1 showed low but detectable levels of mouse FGFR1 on EL08.1D2 stromal cells (Supplementary Fig. 6C). Interestingly, the antibody used for functional inhibition of CD56 had cross-species specificity (not shown), suggesting that blocking of NCAM on stromal cells may contribute to the effect observed by blocking CD56 on NK cells. Taken together, these results show that interactions between human NK cells and EL08.1D2 stromal cells can shape surface expression and subsequent motile behaviour.

### Blocking CD56 perturbs NK cell maturation

**Figure 7:**
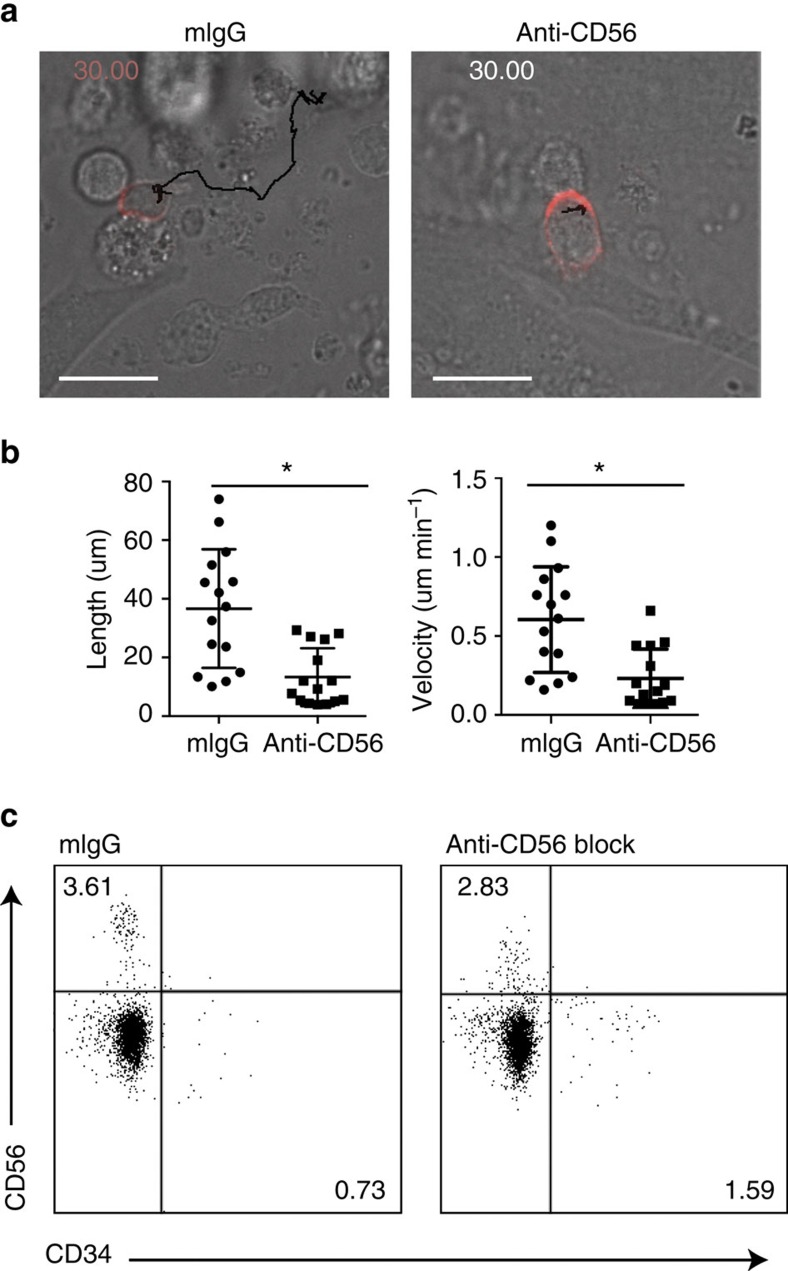
Blocking CD56 ligation throughout development decreases the frequency and motility of mature NK cells. CD34^+^ hematopoietic stem cells were purified from peripheral blood and differentiated *in vitro* to become NK cells in the presence of anti-CD56 blocking antibody or mouse IgG as a negative control as described in Methods. 21 days after the initiation of culture cells were removed from culture and analysed by FACS or incubated on fresh EL08.1D2 stromal cells for 4 h before imaging and cell tracking as in Fig. 1. (**a**) Representative track from mIgG (left) or anti-CD56 (right) treated cultures is shown with its track from 0 to 30 min overlaid; red, CD56. See also Supplementary Movies 7 and 8. Scale bar, 10 μm. (**b**) Cells were tracked as in Fig. 1. *n*=15 from one representative experiment of 3. Data are represented as mean±s.d. *P<0.05 by Student's unpaired *t*-test. (**c**) FACS analysis of NK cell maturation markers from one representative experiment.

To determine whether the increased expression of CD56, which correlated with increased motility during NK cell development, was functionally relevant, we combined the use of CD56-blocking antibody with *in vitro* differentiation of NK cells from CD34^+^ precursors. Blocking antibody was maintained in *in vitro* cultures from the initiation of differentiation. Before imaging, we incubated NK cells derived *in vitro* on fresh stromal cells then imaged and tracked their motility (Fig. 7a; Supplementary Movies 7 and 8). Those NK cells derived in the presence of CD56 blocking antibody showed significantly decreased track length and velocity compared with those in the presence of mIgG as a negative control (Fig. 7b).

Finally, the presence of CD56-blocking antibody from the initiation of *in vitro* differentiation resulted in a decreased frequency of Stage 4 CD56^bright^ NK cells and an increased frequency of CD34^+^ precursors remaining in the culture 21 days after (Fig. 7c). The reduced CD56^bright^ population did show normal levels of expression of other Stage 4 markers (namely CD117, CD62L and CD94), confirming that they were true Stage 4 cells.

## Discussion

The importance of human NK cell development in successful transplant outcomes and immunotherapy is becoming increasingly recognized. The directed shaping of this process is hindered by our lack of understanding of the molecular signalling and intercellular interaction that leads to NK cell development. We sought to resolve these requirements through the use of highly quantitative microscopy while taking advantage of the characterized conditions that lead to *in vitro* generation of mature, functional NK cells. Here we describe the contact-dependent processes leading to human NK cell development, including a structure that we term the developmental synapse.

NK cells have a distinctive migratory phenotype common to many lymphocytes, with a broad leading edge and trailing or elevated uropod^32^,^33^,^34^. Our initial observations of CD56^bright^ NK cells isolated from peripheral blood surprisingly revealed a similar type of migration on EL08.1D2 stromal cells. A notable and unique exception in our model to the previously described standard lymphocytic migratory phenotype was periods of arrest through firm adhesion, particularly seen in the CD56^bright^ subset. Further, we identified this site of attachment as a site of productive signalling, leading to calcium flux and phosphorylated tyrosine residues. Based on these observations we termed this site the developmental synapse. It is unclear whether the NK cell directly converts the uropod to a developmental synapse or whether this is derived from a modified leading edge, as the conventional lytic synapse is (ref. 25). Many of the components found in the uropod are found in the developmental synapse, including F-actin, CD43 and moesin. However, under these conditions it is difficult to delineate a uropod from a developmental synapse by fixed-cell microscopy and further studies are required to definitively distinguish the two. While uropod-like structures have been detected in NK cells undergoing detachment from target cells, these are relatively short-lived (2–3 min)^33^, whereas we detect significant firm adhesion for up to 30 min and failed to visualize cells converting a more conventional appearing synapse (or motility) to a DS. While the developmental synapse may be derived from a uropod, the elements that define an immunological synapse, namely stable adhesion, polarity of F-actin, and signalling, confirm its place as a bona fide immunological synapse.

While CD34^+^ precursors form relatively stable contacts with developmentally supportive stromal cells, human NK cells acquire motility with progressive maturation, which correlates with the expression of CD56 on developing NK cells. The exception to this generalization may be the CD56^dim^ subset, which despite having lower density of CD56 on the surface consistently displays the highest velocity with the least amount of time in arrest. We propose that the purpose of the arrest and tether phenomenon is to seek and signal through the developmental synapse, leading to NK cell maturation. This would be consistent with observed behaviour of mouse NK cells in lymph node, which can be seen to engage in similar intermittent motility^17^,^18^. This behaviour could provide human NK cells with the ability to mature in multiple microenvironments through their ability to seek those signals necessary for progression to the next stage of maturation. Accordingly, it is predicted that CD56^bright^ NK cells would spend a greater time in arrest as opposed to the CD56^dim^ subset, particularly as the CD56^bright^ subset is highly enriched in secondary lymphoid tissue^3^. The reduced frequency of CD56^+^ NK cells following sustained block with anti-CD56 antibody suggests that this mechanism is required for appropriate subset generation. The exact place in which CD56^neg^ NK cell fall into this schema is poorly understood, as their developmental state is not known, however they are proposed to be terminally differentiated or exhausted cells^27^.

CD56 is enriched at our proposed DS, yet it is not seemingly required for adhesion. Experiments with blocking antibody consistently show that adhesion to target cells was maintained, which is consistent with previous reports showing that homotypic adhesion is not mediated between NCAM-expressing hematopoietic lines and human NK cells^12^. Instead, CD56, which is the 140 kDa isoform of NCAM^12^, appears to be required for migration on stromal cells, and thus is consistent with prior studies showing that NCAM-140 mediates motility of glioma cells^13^. Further work is required to elucidate this pathway, however the signalling pathways downstream of NCAM-140 have been well studied^35^. Cell migration and neurite outgrowth are mediated through Src family kinase-dependent activation of focal adhesion kinase^14^. Fixed-cell confocal microscopy showing phospho-tyrosine accumulation at the site of CD56 accumulation at the DS and the effect of the inhibition of Src kinase on cell migration suggest a similar mechanism in NK cells and it will be of interest to determine if Pyk2, the focal adhesion kinase homologue expressed by NK cells, is similarly required. Single-cell analysis of PC12 cells shows that NCAM ligation results in intracellular calcium release and subsequent neurite outgrowth as a result of PLCγ and PKC signalling^36^,^37^, suggesting that these pathways may act downstream of CD56 to mediate release from intracellular calcium stores in NK cells. Alternatively, CD56 may potentiate signals leading to migration through integrins, particularly β1 integrin, as has been previously reported in the case of rat neuroblastoma cells migrating on fibronectin^38^. Finally, it is possible that relative density of CD56 on the cell surface may affect exposure of other existing adhesion receptors or access by adhesion ligands, therefore increasing strength of binding and resulting in decreased motility. The requirement for CD56 both in motility and its subsequent accumulation in the developmental synapse is not clearly understood. Determining the sites of active CD56 signalling, the relationship to F-actin-driven transport and the composition and critical size threshold of CD56-containing clusters, such has been examined for activating and inhibitory receptors in NK cells^39^ and the TCR in T cells^40^, will be an important future direction for this work.

Our data showing that incubation on stroma maintains expression of CD62L demonstrates that interactions with stroma influence expression of adhesion molecules. While not the sole means of adhesion or migration, our data shows a contribution of CD62L to NK cell migratory behaviour, consistent with its previously described role in lymph node trafficking. The unknown ligand on stromal cells that maintains CD62L expression may be analogous to a signal in human lymph node that maintains retention in secondary lymph node tissue through CD62L expression. We did not observe defects in motility on stromal cells in NK cells isolated from LAD-1 patients lacking β2 integrin expression on the cell surface (Mace, unpublished observations). This suggests that LFA-1 is not necessarily required for migration, consistent with similar observations made in T cells^41^. While we observed F-actin at the DS, it was difficult to test a specific role for F-actin in NK cell migration, as depolymerizing F-actin using drugs such as Latrunculin A resulted in depolymerization of F-actin in stromal cells. However, inhibition of Myosin IIA function with blebbistatin abrogated migration (not shown), suggesting that Myosin IIA-mediated F-actin force generation is required, as is the case in T cells undergoing amoeboid motility^42^.

An outstanding question that this work raises is the nature of interactions between human NK cells and murine-derived cell lines, particularly the engagement of CD56. Previous studies have shown that contact with EL08.1D2 stromal cells enhances the efficiency of human NK cell development^5^. This has been further supported by work showing that ligation of CD56 by FGFR1 on human synovial fibroblasts specifically supports the maturation of CD56^bright^ to CD56^dim^ NK cells^9^. While this suggests sites of inflammation as a location for human NK cell development^9^, our study complements this by describing the structure of contacts found throughout the spectrum of human NK cell development. Further, we identify murine NCAM-1, which we show is expressed on stromal cells, as a potential ligand for human CD56. We observed colocalization of the two molecules, supporting the hypothesis that homotypic binding of CD56 to NCAM may be one mechanism by which human NK cells mediate migration and development. In addition, murine FGFR1 is expressed on EL08.1D2 stromal cells, although it is unclear as to whether this is a ligand for human CD56, particularly as the expression is low. While it may be difficult to extend the observations made using a mouse cell line to a human system, it bears remembering that the EL08.1D2 system represents an established and uniquely supportive *in vitro* developmental model for human NK cells and is considered the gold standard for human NK cell generation^4^. Nevertheless, efforts are underway to recapitulate a purely human model of lymphoid development to extend our findings to a more physiologically relevant setting. Similarly, there may be murine molecules playing functionally homologous yet evolutionarily divergent roles to CD56 in a mouse setting, akin to the functional homology observed between murine Ly49 receptors and human KIRs.

In conclusion, we have used high-resolution microscopy to probe the nature of cell–cell interactions during NK cell development. We find that the interactions that drive NK cell maturation are not static and instead progress from stable contacts at the CD34^+^ stage to predominantly motile cells with distinct intermittent arrest through a novel platform that we term the developmental synapse. This motility requires CD56 and the developmental synapse is a site of productive tyrosine and calcium signalling. We propose that NK cell maturation is accompanied by this motile, seeking, mechanism that enables their development in multiple supportive microenvironments. This work defines molecular mechanisms at play in human NK cell development but also identifies a novel paradigm of an organized signalling platform (the developmental synapse) formed between immune cells and stromal cells.

## Methods

### Cells and *in vitro* differentiation from CD34^+^ precursors

NK cells were enriched before cell sorting using RosetteSep (STEMCELLTechnologies) and Ficoll-Paque density gradient centrifugation from routine red cell exchange apheresis performed at Texas Children's Hospital or from healthy normal donors. For the isolation of lymph node NK cells, fresh tonsils were obtained following surgical resection for tonsillectomy at Texas Children's Hospital and single-cell suspensions prepared by first mincing ¼ tonsil segments with a sterile blade in 20 ml PBS, then forcing minced pieces through sterile mesh (using the flat end of a sterile 3 ml syringe) to create a single-cell suspension. The mesh was rinsed with 5 ml of additional PBS then single-cell suspension was layered on 15 ml of Ficoll-Paque in a 50 ml conical tube for density centrifugation at 2,000 r.p.m. for 20 min (no brake). Cells were harvested from the interface and washed with 50 ml PBS by centrifugation at 1,500 r.p.m. for 5 min then resuspended in complete media and counted for downstream applications. All samples were obtained under the guidance and approval of the Institutional Review Board of Baylor College of Medicine and were compliant with the Declaration of Helsinki guidelines.

EL08.1D2 stromal cells were a kind gift from Dr E.A. Dzierzak (Erasmus MC, Netherlands) via Dr J.S. Miller (University of Minnesota, USA) and were maintained on gelatinized plates at 32 °C in 40.5% α-MEM (Life Technologies), 50% Myelocult (STEMCELL Technologies), 7.5% fetal calf serum (Atlanta Biologicals) with β-mercaptoethanol (Gibco, 50 μM l^−1^), Glutamax (Life Technologies, 2 mM), penicillin/streptomycin (Life Technologies, 100 U ml^−1^), and hydrocortisone (Sigma, 10^−6^ M). EL08.1D2 were seeded into 96-well plates then mitotically inactivated by irradiation at 300 rads before the addition of progenitors^43^. *In vitro* NK cell differentiation was performed using previously reported protocols^4^,^43^,^44^. CD34^+^ hematopoietic stem cells were purified to >95% purity by flow cytometric cell sorting and cultured at a density of 2–10 × 10^3^ cells per well on a confluent layer of mitotically inactivated EL08.1D2 stromal cells in Ham F12 media plus DMEM (1:2) with 20% human AB^−^ serum, ethanolamine (50 μM), ascorbic acid (20 mg l^−1^), sodium selenite (5 μg l^−1^), β-mercaptoethanol (24 μM) and penicillin/streptomycin (100 U ml^−1^) in the presence of IL-15 (5 ng ml^−1^) IL-3 (5 ng ml^−1^), IL-7 (20 ng ml^−1^), Stem Cell Factor (20 ng ml^−1^) and Flt3L (10 ng ml^−1^). Media was exchanged every 7 days by replacing 50% of the volume, excluding IL-3 after the first week. For tracking experiments *in vitro* derived cells were removed from culture and incubated on fresh stroma 4 h before imaging in the absence of any cytokines.

For the culture of NK cells and subsequent FACS analysis of CD62L expression, purified NK cells were incubated with or without IL-15 (5 ng ml^−1^) in RPMI media supplemented with 10% fetal calf serum on EL08.1D2 stromal cells or on tissue culture treated plates.

### Flow cytometry and antibodies

Following enrichment purified NK cells were isolated by cell sorting using a BD Aria cell sorter with an 85 μm nozzle at 45 p.s.i. NK cells were incubated with antibodies to CD56 (clone HCD56, AlexaFluor 647 conjugated, Biolegend, 1:100) and CD34 (clone 4H11, PE conjugated, eBioscience, 1:100). Purity after sorting was >95%. For FACS analysis following IL-15 culture and CD34^+^ differentiation, a 10-colour flow cytometry panel was designed (Supplementary Table 1). Flow cytometry was performed on a BD Fortessa. All flow cytometry data analysis was performed with FlowJo (TreeStar Inc.).

### Generation of CRISPR-Cas9 CD56-deleted NK cells

Human NK92 cells^45^ were cultured and maintained in supplemented Myelocult culture media (STEMCELL Technologies) containing 10% penicillin/streptomycin (Life Technologies) and 100 U ml^−1^ IL-2 (Roche). CRISPR-Cas9-mediated deletion of NCAM (CD56) in NK92 was performed by nucleofection of 4 μg of pCMV-Cas9-GFP plasmid DNA containing the guide sequence CGCTGATCTCCCCCTGGCTGGG (Sigma-Aldrich) using the Amaxa nucleofector (Lonza). Forty-eight hours post infection cells were sorted for GFP^+^CD56^−^. CD56 deletion was confirmed by flow cytometry following expansion. For *ex vivo* experiments NK cells were enriched with RosetteSep as described above and the CD56^bright^ population was purified by cell sorting before nucleofection. After 48 h, cells were sorted again for GFP^+^CD56^−^ and confirmed to express other phenotypic markers of NK cells (CD94). Cells were rested overnight before imaging on EL08.1D2 stromal cells.

### Acquisition of microscopy images

For analysis of NK cell motility on stroma, EL08.1D2 stromal cells were grown to a confluent monolayer on Lab-Tek chamber slides pre-coated with 0.1% gelatin (STEMCELL Technologies). Following purification by cell sorting, NK cells were incubated on stromal cells for 4–8 h. For blocking experiments, NK cells were pre-incubated for ten minutes with 20 μg ml^−1^ blocking antibody before addition to stromal cells with anti-NCAM (Millipore) and/or anti-CD62L (DREG-56, Biolegend). For inhibitor experiments, NK cells were pre-incubated with 25 uM PP1 (Tocris Bioscience) or DMSO as vehicle control before addition to stroma cells. Fluorophore-conjugated antibodies (CD56 Alexa Fluor 647, clone H56, Biolegend; CD62L Alexa Fluor 488, Biolegend) were added to chambers (1:50) 15 min before imaging. Live cells in supplemented RPMI media were imaged at 37 °C for 30–120 min imaging every 30 s with the Mark and Find or Tile Scan feature of LASAF software. Images were acquired on a Leica SP8 confocal microscope with 100 × 1.4 numerical aperture (NA) or 63 × 1.4 NA objective. Excitation was provided via a white light laser and detection was with HyD (GaAs) detectors. Images were acquired using LASAF and exported to Volocity or FIJI for data analysis.

For calcium flux experiments, CD56^bright^ NK cells were pre-incubated with 2 μM Indo1 AM (Life Technologies) at 37 °C then washed, incubated on stromal cells or poly-L-lysine coated imaging chambers for 60 min and imaged by live-cell confocal microscopy as described above. Excitation was at 405 nm and emission detection was set to detect from 400 to 550 nm with HyD tunable detectors (Leica).

For fixed-cell microscopy, EL08.1D2 stromal cells were grown to confluence on gelatinized slides as described above. Following purification by FACS sorting (unless otherwise indicated), NK cells were incubated on stromal cells for 3–4 h. Directly conjugated antibodies to surface antigens (CD56, CD62L, 1:50) were added 15 min before fixation and permeabilization with BD Cytofix/Cytoperm. For intracellular staining, fixed cells were incubated with directly conjugated phalloidin to detect F-actin (Alexa Fluor 568, 1:200) or phospho-tyrosine Alexa Fluor 488 (clone 4G10, Biolegend, 1:50). Primary antibody to moesin (clone 38/87, 1:100) was detected with secondary anti-rabbit Alexa Fluor 488 (Biolegend, 1:200) and biotinylated anti-CD43 (clone DF-T1, Miltenyi, 1:100) was detected with streptavidin Alexa Fluor 488 (Biolegend, 1:200). Slides were mounted with ProLong Gold (Life Technologies) and imaged by confocal microscopy using the Leica SP8 confocal microscope described above.

### Image analysis

Tracking of live cells was done using the manual tracking feature in Volocity or FIJI. Tracks were plotted using the Chemotaxis plugin of FIJI. Cells that were in the field of imaging for fewer than two frames were discarded, as were cells which were non-adherent or floating. EL08.1D2 cells were used as de facto fiducial markers to ensure that neither they or the microscope stage was drifting and causing apparent NK cell movement. Length and displacement measurements were derived directly from tracked cells and graphed using GraphPad software. Velocity data was obtained by dividing the total track length by the time of imaging. For analysis of NK–NK conjugates, cells which were conjugated for the entire time of imaging are scored as 100%, while those that became conjugated while imaging and were still conjugated at termination were from the time of conjugation until the end. For arrest coefficient analysis, cells were tracked by manual tracking in FIJI with the tracking point placed at the point where the uropod meets the cell body. Tracks <0.5 μm in length between consecutive time points were considered in arrest and the relative number of time points in which a single cell was found in arrest was normalized to the total number of time points imaged. For aspect ratio analysis, cell length was divided by cell width at the widest point. To measure accumulation of fluorescent signals, mean fluorescent intensity above that of a set background was measured using Volocity and graphed using GraphPad software. Where applicable, the contribution of fluorescence from the stromal cell and baseline fluorescence from the NK cell were subtracted by measuring intensity at unconjugated points and subtracting the intensities of each from the intensity at the synapse^46^. For analysis of polarization, the opposite side from the DS was defined as the midpoint of the distal edge of the cell relative to the point of attachment. Tile scan images of NK92 CD56-KO cells were tracked automatically with Imaris (Bitplane) and exported to GraphPad for graphing and statistics. Tile scan images of Ca^2+^ flux in CD56^bright^, CD56^dim^ and CD56^neg^ NK cells were exported to Volocity for analysis. Cells were identified using the ‘Find Objects' feature and mean intensity of 485 and 530 nm channels was measured as a ratio (485/530) for NK cells on stromal cells and poly-L-lysine coated surfaces. The mean of poly-L-lysine values was obtained for each subset (CD56^bright^, CD56^dim^ and CD56^neg^) and individual values for NK cells on stromal cells were divided by this mean to determine normalized Ca^2+^ flux.

### Western blotting

1 × 10^7^ EL08.1D2 stromal cells were lysed, separated by 4–12% SDS–PAGE gel, transferred to nitrocellulose membrane and then blocked with skim milk. FGFR1 was detected with a mouse monoclonal anti-FGFR1 (clone M19B2, Abcam, 1:750) and actin with anti-actin (Sigma, 1:4,000) followed either by a goat anti-mouse 700 (1:10,000) or a goat anti-rabbit 800 (1:10,000). Purified mouse IgG1κ (clone MOPC-21, Biolegend) was used as an isotype control at the equivalent concentration as anti-FGFR1. Proteins were detected on a LiCor Odyssey.

### Statistics

Ordinary one-way ANOVA was used to compare grouped samples with *post hoc* analysis by Tukey's comparison. Where indicated, Student's unpaired *t*-test was used to compare two conditions. Rayleigh vector test was used to test directionality of migration. For all tests *P*<0.05 was considered significant.

### Data availability

The authors declare that all data supporting the findings of this study are available within the article and its supplementary files or from the authors upon a reasonable request.

## Additional information

**How to cite this article:** Mace, E. M. *et al*. Human NK cell development requires CD56-mediated motility and formation of the developmental synapse. *Nat. Commun.* 7:12171 doi: 10.1038/ncomms12171 (2016).

## Supplementary Material

Supplementary Information Supplementary Figures 1-6, Supplementary Table 1

Supplementary Movie 1 CD56bright on EL08.1D2 stroma.

Supplementary Movie 2 CD56dim on EL08.1D2 stroma.

Supplementary Movie 3 CD56neg on EL08.1D2 stroma.

Supplementary Movie 4 CD56bright NK cell tethered on EL08.1D2 stroma.

Supplementary Movie 5 NK92 on EL08.1D2.

Supplementary Movie 6 NK92 CD56-KO on EL08.1D2.

Supplementary Movie 7 NK cell derived in vitro from CD34+ cell with mIgG.

Supplementary Movie 8 NK cell derived in vitro from CD34+ cell with anti-CD56 blocking
