# Supplementary material for: Human NK cell development requires CD56-mediated motility and formation of the developmental synapse
Source: Nat Commun. 2016 Jul 20;7:12171. doi: 10.1038/ncomms12171 (PMC4961740; doi:10.1038/ncomms12171)
Supplement: Supplementary Information — Supplementary Figures 1-6, Supplementary Table 1 [file ncomms12171-s1.pdf]

Supplementary Figure 1

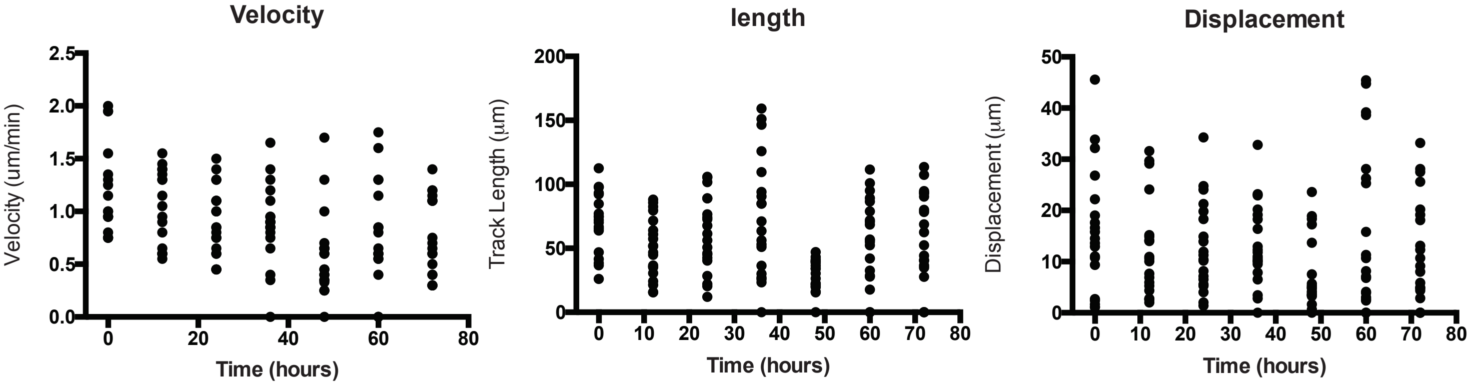

Supplementary Figure 1. A) Effect of incubation on track length, displacement and velocity. CD56^{bright} NK cells were sorted from peripheral blood and incubated for indicated times (X-axis) prior to imaging every 30 seconds for 30-60 minutes on a Leica SP8 laser scanning confocal microscope using a 63X 1.4 NA objective. Cells were tracked using manual tracking in FIJI. n=17-20 per condition.

Supplementary Figure 2

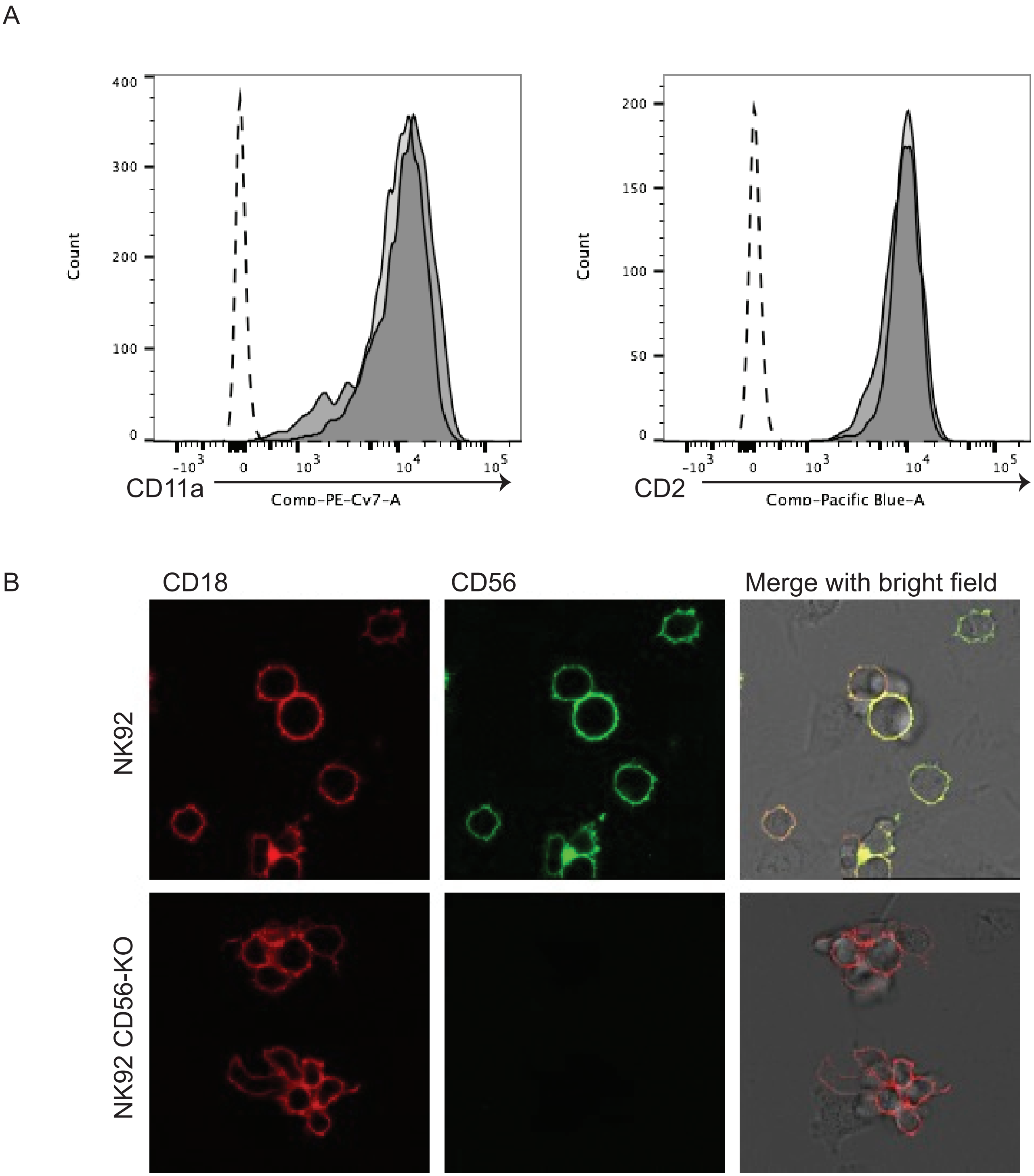

Supplementary Figure 2. Surface expression of adhesion receptors and CD56 on NK92 CD56-KO cells. A) Expression of CD11a (left) and CD2 (right) on NK92 CD56-KO cell lines was measured by flow cytometry. Isotype control is shown as a dashed line, wild-type NK92 are represented by the dark histogram and CD56-KO is the light histogram. B) WT NK92 (top) and CD56-KO (bottom) were stained for CD18 (red) and CD56 (green). Representative of three experiments.

Supplementary Figure 3

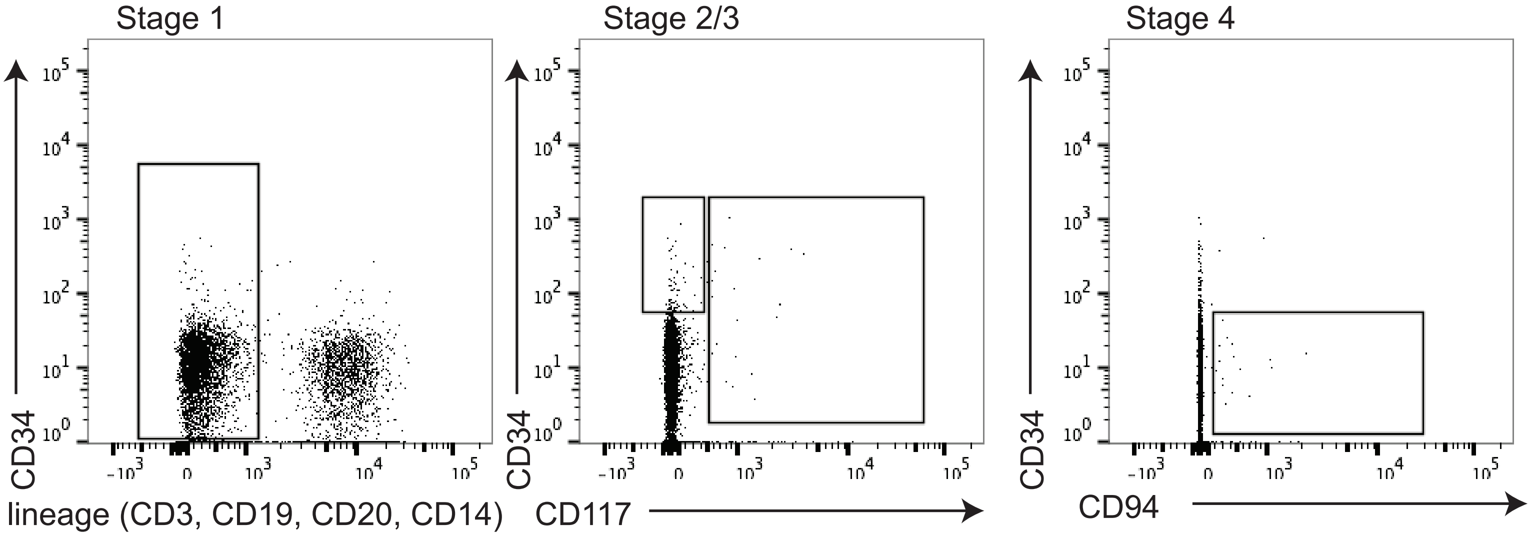

Supplementary Figure 3. Flow cytometry gating strategy for sorting from tonsils. Human tonsils were processed as described in Materials and Methods. Cells were gated on lineage negative and then sorted for $CD34^{+}$ $CD117^{-}$ $CD94^{-}$ (Stage 1), $CD34^{-}$ $CD117^{+/-}$ $CD94^{-}$ (Stage 2-3) and $CD34^{-}$ $CD117^{+/-}$ $CD94^{+}$ (Stage 4) as shown. Shown is one representative of three experiments.

Supplementary Figure 4

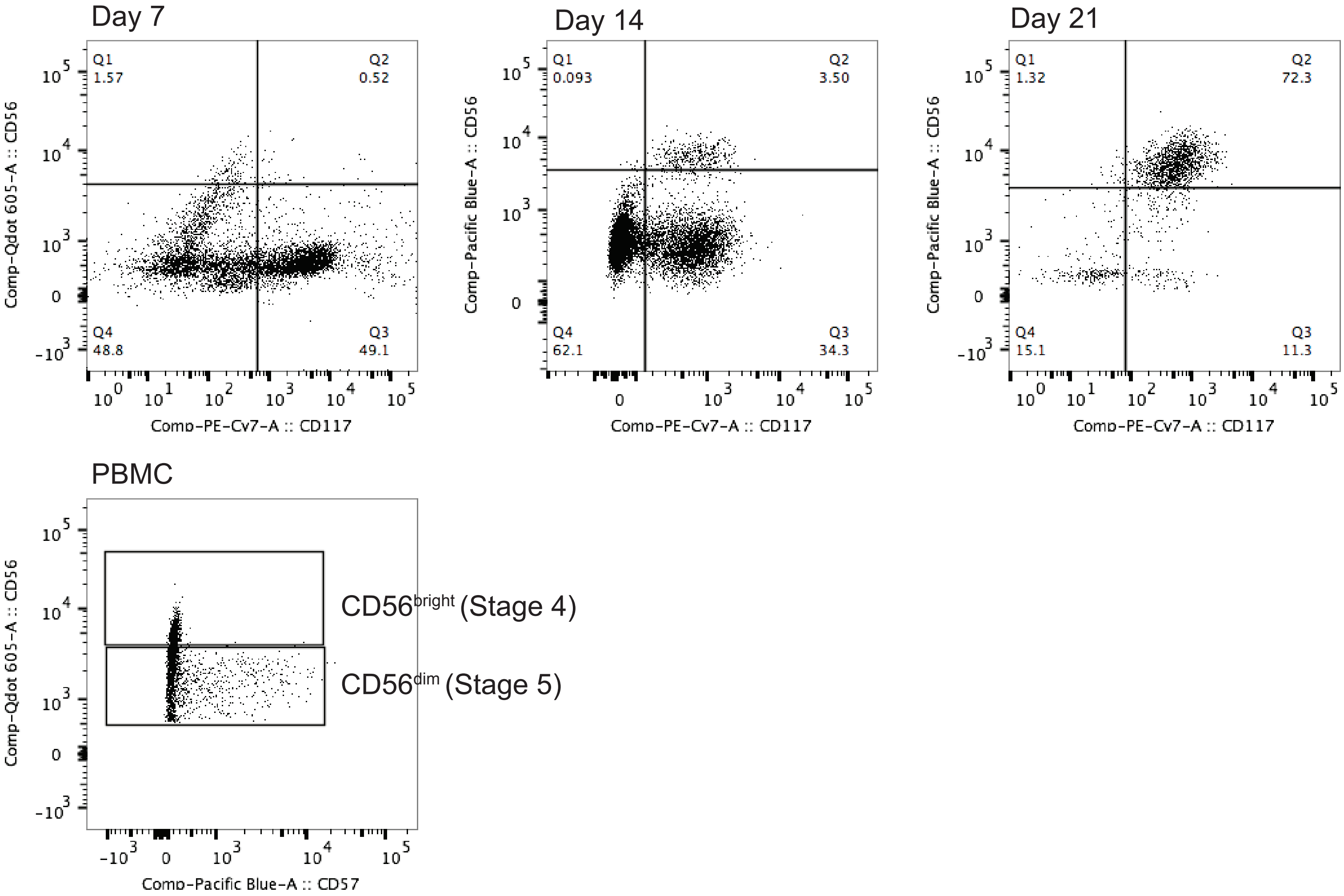

Supplementary Figure 4. Flow cytometry analysis of CD56 and CD117 expression throughout in vitro differentiation. In vitro derived NK cells were harvested at time points indicated and evaluated by flow cytometry for markers of interest including CD56 and CD117 (top). CD56 expression fell within the CD56^{bright} compartment as determined by comparison with PBMC from the same donor, where CD57 was used to clearly demarcate CD56^{bright} from CD56^{dim} (bottom panel). Shown is one representative of three experiments.

Supplementary Figure 5

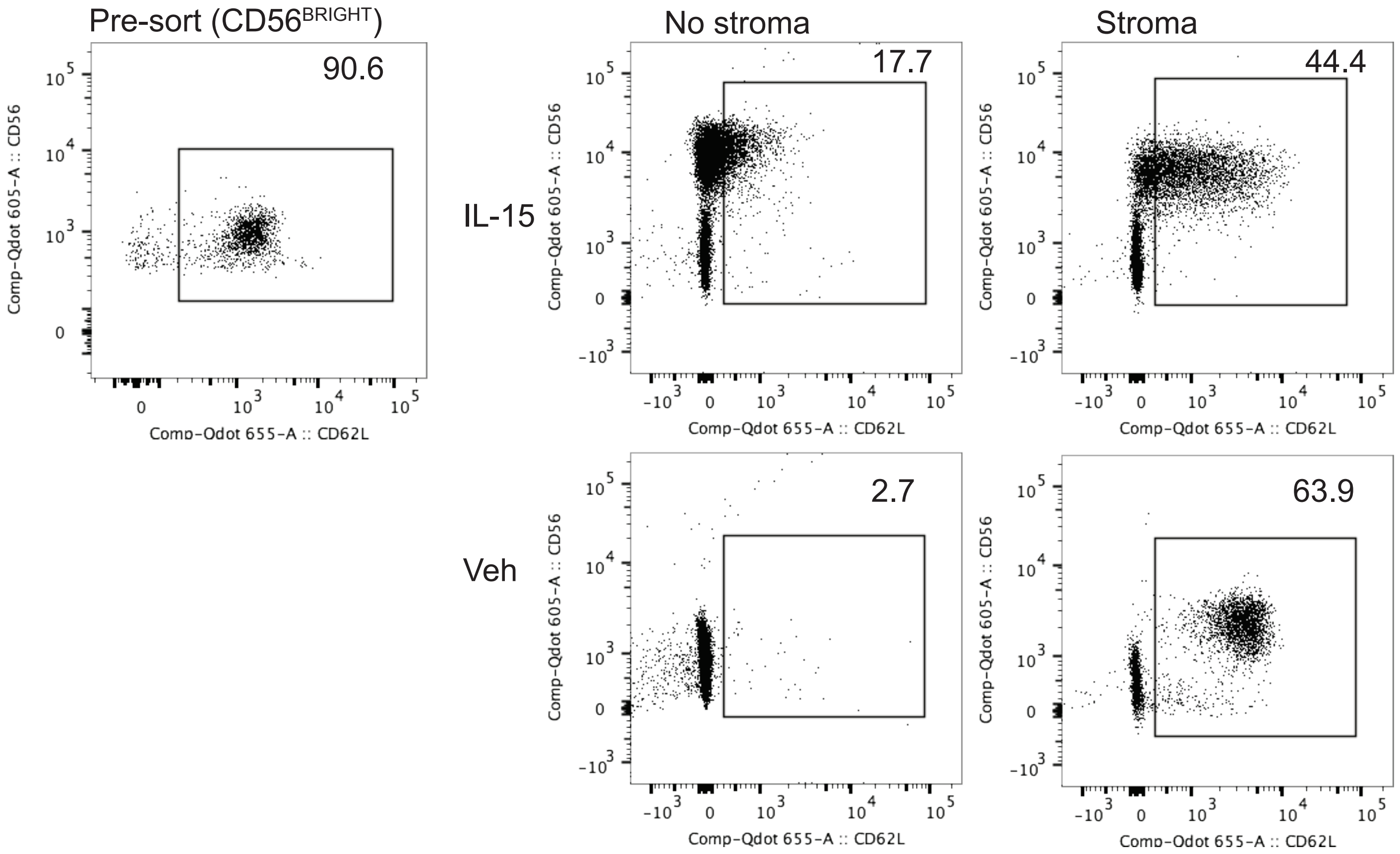

Supplementary Figure 5. CD62L expression on mature NK cells is reduced in the absence of EL08.1D2 stromal cells. CD56^{bright} NK cells were purified and incubated on EL08.1D2 stromal cells or in uncoated tissue culture dishes for 3 days in the presence of 5 ng/ml IL-15 or vehicle control. Representative FACS plots are shown from one experiment in which cells were analyzed by multiparametric flow cytometry for markers of NK cell maturation.

Supplementary Figure 6

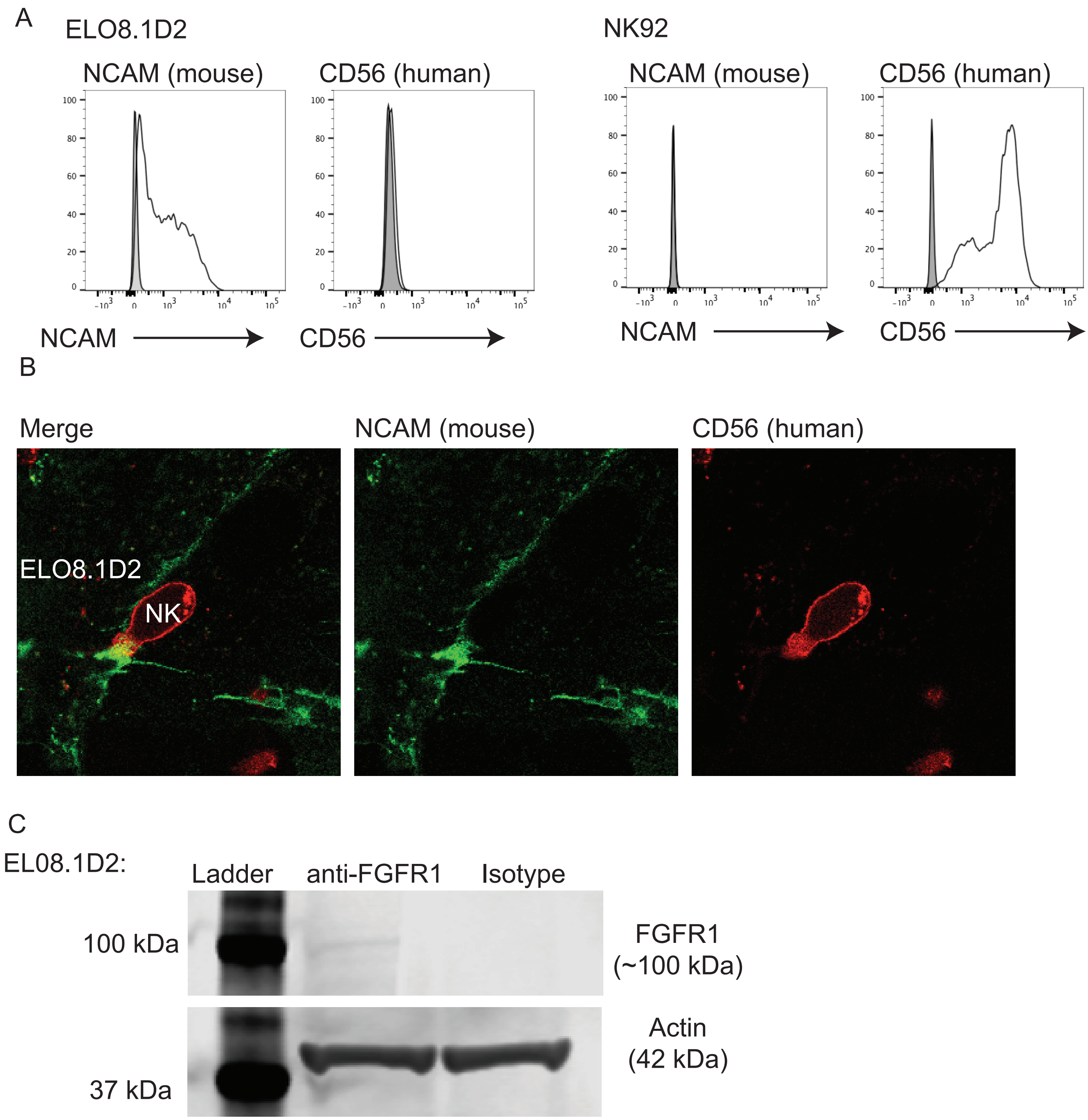

Supplementary Figure 6. EL08.1D2 stromal cells express previously identified ligands for CD56. A) EL08.1D2 stromal cells (top) or the NK92 cell line (bottom) were stained with anti-CD56 (human, clone HCD56) or anti-NCAM (mouse, clone 809220) (open histograms) and appropriate isotype control (grey histograms) as indicated. B) EL08.1D2 stromal cells were pre-incubated with anti-NCAM (mouse, clone 809220) and human NK cells labeled with anti-CD56 (HCD56) were conjugated for 60 min. Images were acquired by confocal microscopy. C) EL08.1D2 stromal cell lysates were probed for anti-FGFR1 (top, right; clone M19B2) or with anti-mouse IgG1 isotype control (top, left; clone MOPC-21) and probed in parallel with anti-actin (bottom) as a loading control. Results shown are representative of 3 independent experiments.

Supplementary Table 1: flow cytometry antibodies

**Fluorophore**	**Marker**	**Clone; Source; Dilution**
APC	CD94	DX22, Biolegend; 1:100
APC Cy7	CD34	581, Biolegend; 1:100
Pacific Blue	CD57	NC1, Beckman Coulter, 1:100
QD605	CD56	HCD56, Biolegend, 1:200
QD655	CD62L	DREG56, Biolegend, 1:100
QD711	CD3	SK7, Biolegend, 1:200
FITC	Perforin	δG9, BD Pharmingen, 1:20
PE Cy7	CD117	104D2, Biolegend; 1:10
CF 594	CD16	3G8, Biolegend, 1:300
